# Ultrafast Photochemical Dynamics of Dinitrosyl Iron Complexes Investigated by Femtosecond Time-Resolved Infrared Spectroscopy

**DOI:** 10.3390/ijms26188835

**Published:** 2025-09-10

**Authors:** Hojeong Yoon, Juhyang Shin, Seongchul Park, Manho Lim

**Affiliations:** 1 Department of Chemistry and Chemistry Institute for Functional Materials, Pusan National University, Busan 46241, Republic of Korea; yun6793@naver.com (H.Y.); dksvmflzk@gmail.com (J.S.); papagenona@gmail.com (S.P.); 2 Korea Institute for Future Earth, Pusan National University, Busan 46241, Republic of Korea

**Keywords:** dinitrosyl iron complex (DNIC), photoexcitation dynamics of DNIC, mononitrosyl iron complex (MNIC), reaction dynamics in solution, 2-mercaptoethanol-ligated DNIC, photoactivated NO^−^-releasing compound, femtosecond vibrational spectroscopy

## Abstract

Dinitrosyl iron complexes (DNICs) are the most abundant nitric oxide (NO) metabolites in NO-producing cells and can be used as a platform for photochemical vehicles for NO donors. However, not much is known about the photochemical dynamics of DNICs. This study investigates the photoexcitation dynamics of a mononuclear DNIC ligated with 2-mercaptoethanol, [(HOCH_2_CH_2_S)_2_Fe(NO)_2_]^−^, in D_2_O solution through femtosecond infrared spectroscopy. Approximately 70% of the excited [(HOCH_2_CH_2_S)_2_Fe(NO)_2_]^−^ at 400 nm relaxes back to the ground state with a time constant of 270 ps, and the remaining dissociates NO^−^ with a time constant of 630 ps. The resulting mononitrosyl iron complex, [(HOCH_2_CH_2_S)_2_Fe(NO)(D_2_O)_2_], formed by a rapid coordination of D_2_O molecule to the nascent photoproduct, [(HOCH_2_CH_2_S)_2_Fe(NO)], reacts with the abundant thiolate, HOCH_2_CH_2_S^−^, in solution, producing [(HOCH_2_CH_2_S)_3_Fe(NO)]^−^ with a rate constant of 1.3 × 10^7^ M^−1^s^−1^. The detailed photochemical dynamics described herein lays the groundwork for the development of NO^−^ donors using DNICs with controlled and tunable photoreactivity for potential therapeutic applications.

## 1. Introduction

Nitric oxide (NO) is a crucial signaling molecule involved in various physiological processes, such as vasodilation, neurotransmission, and immune system response. Its biological activity arises from its ability to interact with the metal centers of various biological molecules, particularly those of iron–sulfur clusters in proteins [1]. One such interaction product is the dinitrosyl iron complex (DNIC), a coordination compound with a Fe(NO)_2_ unit essential for NO metabolism and storage [2]. DNICs are spontaneously generated in cells and are the most abundant NO-derived cellular adducts [3,4]. Iron–sulfur clusters in proteins typically degrade upon exposure to NO, producing both mononuclear and dinuclear DNICs, also called Roussin’s red ester (RRE) [5,6,7,8]. Thiolate ligated mononuclear DNICs, [Fe(NO)_2_(RS)_2_]^−^, derived from abundant cellular ligands, such as cysteine residues or glutathione, are important for the regulation of cell redox and NO bioavailability [9,10,11].

DNICs participate in diverse physiological functions and act as a natural storage and transport system for NO [1,12]. Because the release of NO from DNICs can be utilized in the therapeutic applications of NO, a large number of synthetic efforts have been made to tune the NO-releasing properties of DNICs by designing suitable ligands [1,12]. However, even though DNICs are crucial for multiple biological processes, their photochemical properties have remained relatively unexplored. Given their potential role as the donors and biological sensors of NO, it is critical to understand the dynamics of DNICs under photoexcitation [10,13,14,15]. The challenge, however, lies in experimentally capturing the rapid photochemical processes, as the photochemical reactions of DNICs can occur on femtosecond to picosecond timescales. The real-time photochemical dynamics of DNICs can be investigated by examining the vibrational modes of intermediates and products through femtosecond time-resolved infrared (TRIR) spectroscopy, which could reveal the pathways and mechanisms through which DNICs undergo photoinduced transformations.

Mononuclear DNICs share a population balance with RRE, depending on the pH and thiol concentration in the solution. High pH levels and thiol concentrations promote the formation of mononuclear DNICs, while low pH levels and thiol concentrations promote the formation of RRE [16]. According to the Enemark–Feltham notation, mononuclear DNICs can be represented as [Fe(NO)_2_]^9^, where two NO ligands bind to a d^7^ iron center. This configuration leads to a net charge of −1 and an overall multiplicity of doublet, as confirmed by electron paramagnetic resonance (EPR) studies [11,17]. Because NO acts as a redox non-innocent ligand, several views have been proposed regarding the electronic structures of mononuclear DNICs, i.e., {Fe^I^(NO^•^)_2_}^9^, {Fe^II^(NO^•^)(NO^−^)}^9^, and {Fe^III^(NO^−^)_2_}^9^, as well as their resonance hybrids [18,19,20,21].

Previous studies have reported the release of NO^−^ or NO from mononuclear DNICs through photolysis or heat resulting from the NO^−^ or NO transfer from bidentate neutral mononuclear DNICs to the Fe^III^ or Fe^II^ complex, respectively [1,22,23]. In contrast, a previous study on the nanosecond photolysis of the cysteine-ligated DNIC reported that mononuclear DNICs do not dissociate NO or thiolate upon photoexcitation, although photoexcited RRE causes reversible NO dissociation [16,24,25]. A previous study speculated the dissociation of the cysteine radical based on the photoexcited cysteine-ligated mononuclear DNIC [16].

**Figure 1:**
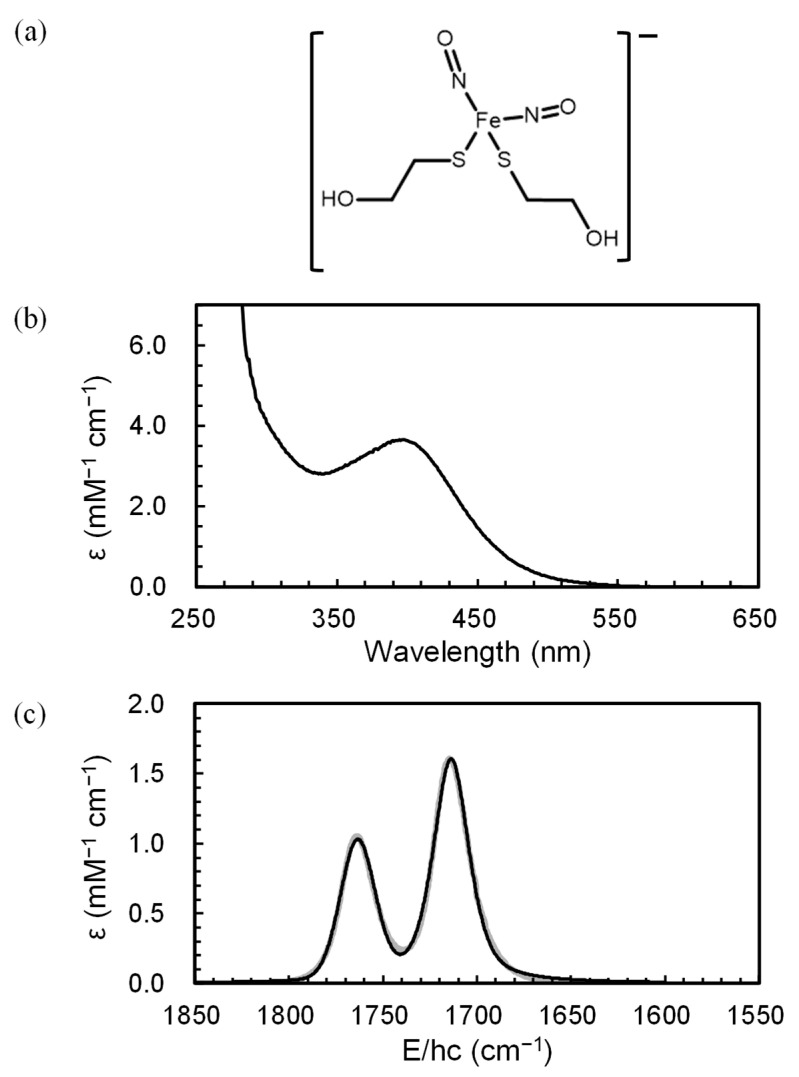
(**a**) Molecular structure of [Fe(NO)_2_(MerS)_2_]^−^ and its equilibrium (**b**) UV-Vis and (**c**) mid-IR absorption spectrum in D_2_O solution. The vibrational spectrum (thick gray line), composed of two bands arising from the symmetric and asymmetric NO stretching modes, is well reproduced by sum of two Voight profiles peaked at 1764 and 1714 cm^−1^ (thin black line).

This study aims to explore the photodynamics of mononuclear DNICs using the simplest water-soluble DNICs—a 2-mercaptoethanol-ligated DNIC, [(HOCH_2_CH_2_S)_2_Fe(NO)_2_]^−^, denoted as [Fe(NO)_2_(MerS)_2_]^−^ (Figure 1a). The simplicity of this system allowed us to focus on the core photochemical processes without interference from more complex ligands. The photoexcitation dynamics of [Fe(NO)_2_(MerS)_2_]^−^ in D_2_O solution after excitation at 400 nm was investigated using femtosecond TRIR spectroscopy at 293 K. Dissociation of NO^−^ from [Fe(NO)_2_(MerS)_2_]^−^ upon excitation at 400 nm and the subsequent reaction of the nascent photoproducts were probed, which provided new insights into the photochemical behavior of mononuclear DNICs.

## 2. Results and Discussion

To study the ultrafast photochemical kinetics of [Fe(NO)_2_(MerS)_2_]^−^, we first characterized the ground state of the DNIC by UV-Vis and IR spectroscopy and then by TRIR spectroscopy after photoexcitation. The equilibrium UV-Vis spectrum of [Fe(NO)_2_(MerS)_2_]^−^ in D_2_O (Figure 1b) exhibited a prominent absorption peak at 394 nm, a characteristic peak for the mononuclear DNIC [16,26,27,28,29,30]. The FTIR spectrum of [Fe(NO)_2_(MerS)_2_]^−^ in D_2_O was recorded. The spectral region for the NO stretching modes is shown in Figure 1c. Two characteristic NO stretching vibrations were observed. According to the density functional theory (DFT) calculations conducted using the TPSSh functional with the def2SVP basis set (see Section 3.3), the weaker band at 1764 cm^−1^ arises from the symmetric NO stretching mode and the stronger band at 1714 cm^−1^ from the asymmetric NO stretching mode. These vibrational modes are essential markers for tracking changes in the DNIC structure during the photochemical cycle. Specifically, the shift of these peaks upon photoexcitation can provide evidence for changes in the electronic environment around the NO ligands that can result in the dissociation of NO from the complex. These assignments can be used as a reference for interpreting the transient spectra observed in TRIR experiments.

**Figure 2:**
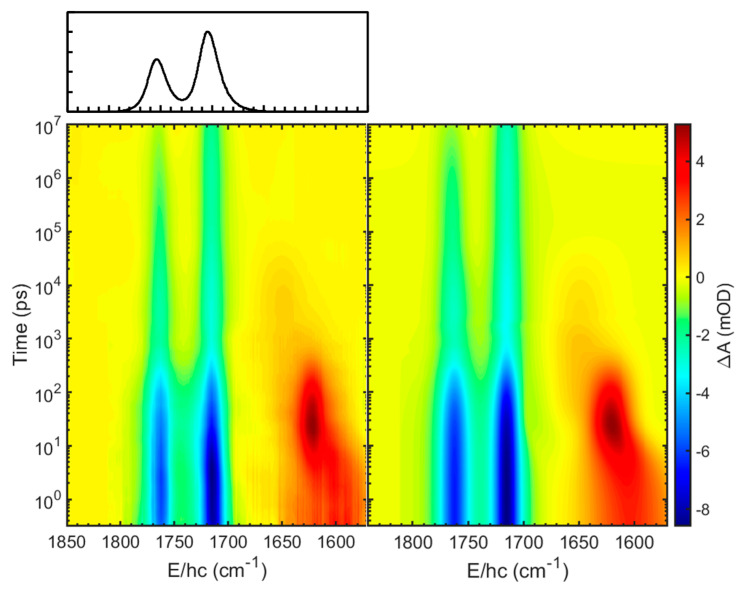
Two-dimensional contour map of the time-resolved infrared (TRIR) spectra of [Fe(NO)_2_(MerS)_2_]^−^ in D_2_O at 293 K after excitation at 400 nm (**left panel**) and its fit (**right panel**) using four basis spectra shown in Figure 4a (see text). The equilibrium FT-IR spectrum is also shown above the left panel. The absorbance difference (ΔA) was obtained by subtracting the pre-photoexcitation absorbance from post-photoexcitation absorbance. Absorbance is reported in optical density (OD) units, where 1 mOD = 10^−3^ OD. The x-axis label is the wavenumber, represented by energy (E) divided by Planck’s constant (h) and the speed of light (c).

**Figure 3:**
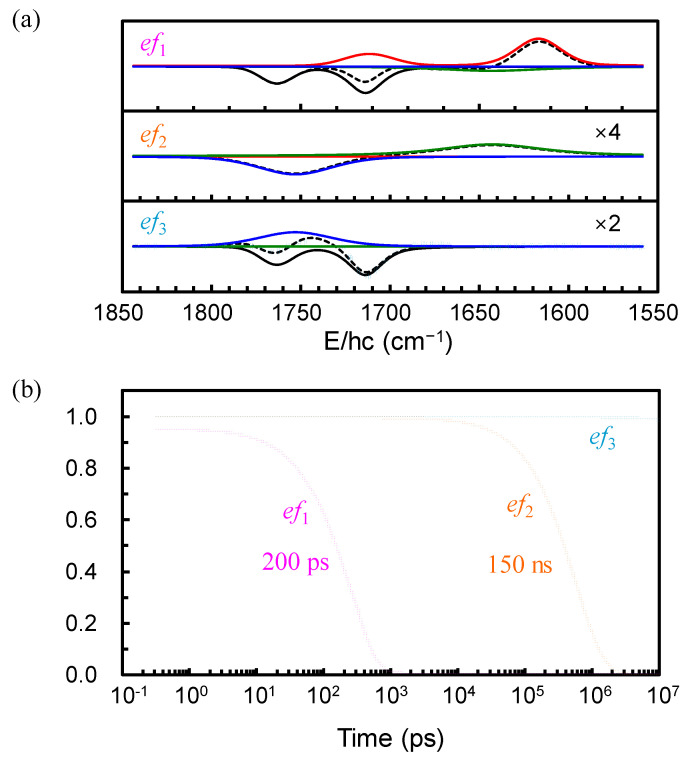
(**a**) Eigenfunctions (colored open circles; denoted to *ef*_1_, *ef*_2_, and *ef*_3_) obtained by the singular value decomposition (SVD) analysis of the time-resolved infrared (TRIR) spectra of [Fe(NO)_2_(MerS)_2_]^−^ in D_2_O at 293 K after excitation at 400 nm. Eigenfunctions are decomposed into color-coded basis spectra shown in Figure 4a to globally fit the TRIR spectra (see text). Eigenfunctions are well described by the sum (dashed black lines) of the basis spectra (red, green, blue, and black lines). Eigenfunctions *ef*_2_ and *ef*_3_ are expanded 4 and 2 times, respectively, for a better view. (**b**) Kinetics of the eigenfunctions for the SVD analysis of the TRIR spectra. Decay time constants are also shown.

**Figure 4:**
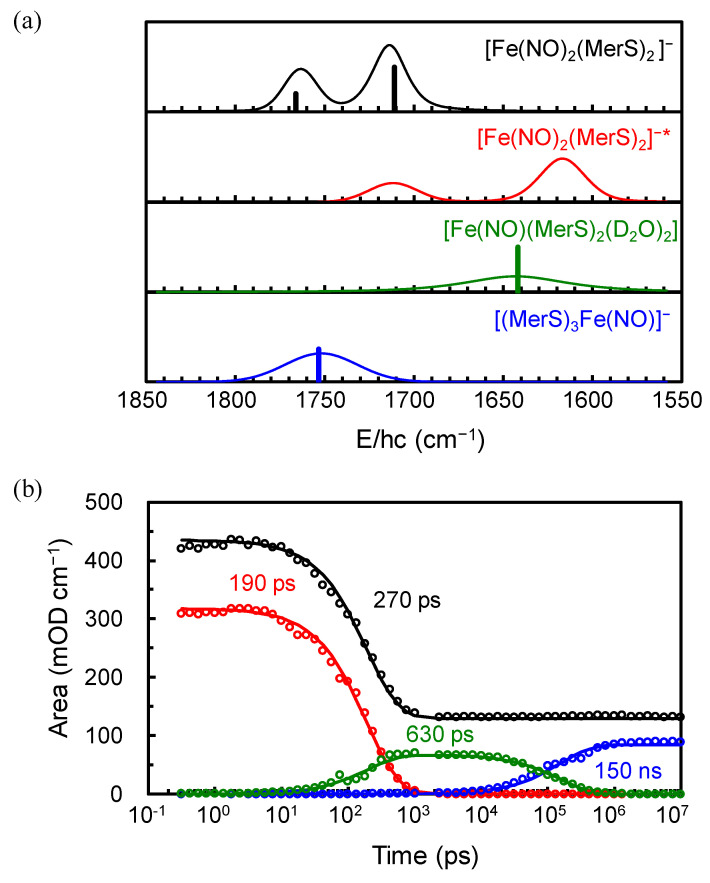
(**a**) Basis spectra used to globally fit the time-resolved infrared (TRIR) spectra of [Fe(NO)_2_(MerS)_2_]^−^ in D_2_O at 293 K after excitation at 400 nm. The basis spectra were determined by decomposing three eigenfunctions obtained from the SVD analysis of the TRIR spectra (see text). The basis spectra were assigned by combining the calculated vibrational spectra with the plausible kinetic scheme (see text). The calculated vibrational spectra of the corresponding compounds are shown as sticks with scaling factors of 0.91–0.967. (**b**) Time-dependent amplitude changes of the basis spectra of [Fe(NO)_2_(MerS)_2_]^−^ (black open circles), [Fe(NO)_2_(MerS)_2_]^−*^ (red open circles), [Fe(NO)_2_(MerS)_2_(D_2_O)_2_] (green open circles), and [Fe(NO)(MerS)_3_]^−^ (blue open circles) fitting TRIR spectra of [Fe(NO)_2_(MerS)_2_]^−^ in D_2_O at 293 K after excitation at 400 nm. The amplitude represents the integrated area of the absorption bands in the probed spectral region. Black circles represent the amplitude of the bleach and the other circles those of absorption bands. The amplitude changes were fit with exponential decay and/or growth functions and the time constants are shown.

**Figure 5:**
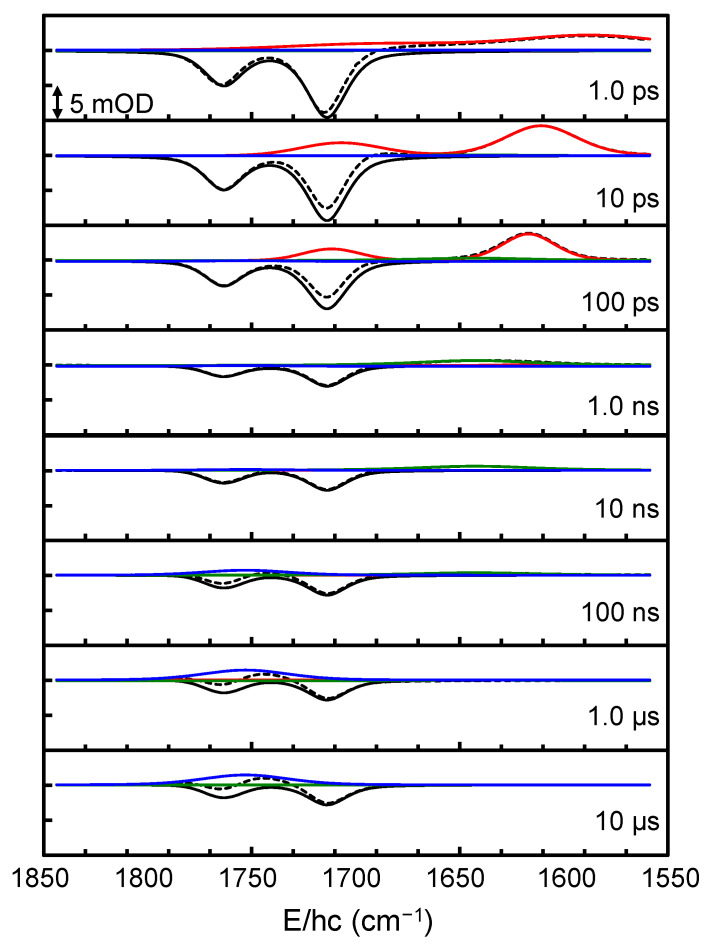
Representative time-resolved infrared (TRIR) spectra of [Fe(NO)_2_(MerS)_2_]^−^ in D_2_O at 293 K after excitation at 400 nm. Data (open circles) are well described by the sum (dashed black lines) of four basis spectra: the spectra of [Fe(NO)_2_(MerS)_2_]^−^ (black solid lines), [Fe(NO)_2_(MerS)_2_]^−*^ (red solid lines), [Fe(NO)(MerS)_2_(D_2_O)_2_]^−^ (green solid lines), and [Fe(NO)(MerS)_3_]^−^ (blue solid lines), shown in Figure 4a (see text).

The TRIR spectra were collected after the excitation of [Fe(NO)_2_(MerS)_2_]^−^ in D_2_O at 293 K using 400-nm pulse in the broad time range from 0.3 ps to 10 μs comprising the spectral region of 1850–1550 cm^−1^. This spectral region was selected because it allows one to focus on the characteristic NO stretching modes of the DNIC species, which appear without any interference from the solvent absorption bands. The 2D contour map of the TRIR spectra is shown in the left panel of Figure 2. The TRIR spectra exhibit immediate bleach (negative-going signal) at the positions of the equilibrium absorption bands of [Fe(NO)_2_(MerS)_2_]^−^, implying that the population of the ground state of [Fe(NO)_2_(MerS)_2_]^−^ is depleted within 0.3 ps, the earliest time in the current experiment, due to the photoexcitation and/or photodissociation of the excited [Fe(NO)_2_(MerS)_2_]^−^. The immediate appearance of new absorption features in the region of 1680−1550 cm^−1^, which evolve with time, also indicates that the excited-state and/or new compounds appear within 0.3 ps after excitation and undergo further reaction. These new absorptions were initially much broad, then shifted to blue, and finally settled at 1712 and 1617 cm^−1^ with a time constant of 5.5 ps (vide infra). The narrowing and blue-shifting of the transient absorptions arises from the thermal relaxation of the initially hot species produced upon immediate relaxation of the excited species [25]. The 400-nm photon excites [Fe(NO)_2_(MerS)_2_]^−^ to its metal-to-ligand charge transfer (MLCT) state [31]. This MLCT transition involves an electron transfer from the iron center to the NO ligands, resulting in a red-shift of the NO stretching frequencies due to increased electron density on the antibonding orbitals of the NO ligands. Therefore, both the new absorptions at 1712 and 1617 cm^−1^ were assigned to the NO stretching modes of [Fe(NO)_2_(MerS)_2_]^−^ in the excited (MLCT) state, i.e., [Fe(NO)_2_(MerS)_2_]^−*^. [Fe(NO)_2_(MerS)_2_]^−*^ appears to release excess energy thermally with a time constant of 5.5 ps before it undergoes photoreaction and/or electronic relaxation to the ground state. Initially, the TRIR spectra were analyzed using the singular value decomposition (SVD) to obtain the eigenfunctions involved in the TRIR spectra [32,33]. As shown in Figure 3, the TRIR spectra involve three eigenfunctions (*ef*_1_, *ef*_2_, and *ef*_3_). The eigenfunctions *ef*_1_ and *ef*_2_ decay with time constants of 200 ps and 150 ns, respectively, while *ef*_3_ maintains its magnitude up to 10 μs, the latest time in the current experiment. The positive features in the eigenfunctions of the SVD are related to the decaying spectra and the negative features to the growing spectra [33]. The TRIR spectra bleach because of the population depletion of the ground state of [Fe(NO)_2_(MerS)_2_]^−^; consequently, the inverted equilibrium spectrum should appear in the eigenfunctions when the ground-state population recovers. When the three eigenfunctions of SVD analysis are combined with the equilibrium spectrum and the calculated vibrational spectra for the expected compounds in the photochemical process of [Fe(NO)_2_(MerS)_2_]^−^, the basis spectra were found to fit the TRIR spectra [32]. Four basis spectra shown in Figure 4a, one of which is the equilibrium spectrum of [Fe(NO)_2_(MerS)_2_]^−^, were obtained to globally fit the TRIR spectra [32,33]. As shown in Figure 3a, the eigenfunctions of the SVD analysis can be described by the combination of the basis spectra of the TRIR spectra shown in Figure 4a. Specifically, the eigenfunction *ef*_1_ decaying with a time constant of 200 ps is a combination of three basis spectra: one basis spectrum with two absorption bands, one each at 1712 and 1617 cm^−1^, as a decaying basis spectrum (a positive feature); another with a broad absorption band at 1643 cm^−1^ as a growing basis spectrum (a negative feature); and the third is the equilibrium spectrum of [Fe(NO)_2_(MerS)_2_]^−^ as a growing basis spectrum (a negative feature). The eigenfunction *ef*_2_ decaying with a time constant of 150 ns shows that the basis spectrum with an absorption band at 1753 cm^−1^ grows (a negative feature) while that with an absorption band at 1643 cm^−1^ decays (a positive feature). The eigenfunction *ef*_3_ is a combination of the basis spectrum with an absorption band at 1753 cm^−1^ (positive feature) and the equilibrium spectrum of [Fe(NO)_2_(MerS)_2_]^−^ (negative feature). As shown in Figure 2 and Figure 5, the TRIR spectra were well reproduced by the sum of four basis spectra shown in Figure 4a.

Time-dependent amplitude changes of the basis spectra were obtained from the global fitting of the TRIR spectra (Figure 4b). Absorption bands for the excited state decay with a time constant of 190 ps, the bleach bands recover with a time constant of 270 ps, and the band at 1643 cm^−1^ grows with a time constant of 630 ps. These results imply that the excited molecule branches into the ground state and the compound with the band at 1643 cm^−1^. Approximately 70% of the bleach signal recovers, while the remaining 30% maintains its amplitude up to about 10 μs. The basis spectrum with the band at 1643 cm^−1^ decays, while that with the band at 1753 cm^−1^ grows with a time constant of 150 ns. Consequently, approximately 70% of the excited [Fe(NO)_2_(MerS)_2_]^−^ relaxes into the ground state with a time constant of 270 ps, while the remaining 30% undergoes a photochemical reaction producing a new compound with the band at 1643 cm^−1^ with a time constant of 630 ps. This new compound undergoes further reaction producing another compound with the band at 1753 cm^−1^ with a time constant of 150 ns.

Among the four basis spectra, two have two absorption bands each with stronger absorption at the lower frequency, one of which is the equilibrium spectrum; the remaining two basis spectra have one absorption band each. The calculated vibrational spectra for various compounds expected from the photoexcitation of [Fe(NO)_2_(MerS)_2_]^−^ reveal that compounds with two NO ligands (DNIC) have two absorption bands—one corresponding to the symmetric NO stretching mode and the other to the asymmetric NO stretching mode with stronger absorption in the asymmetric mode at the lower frequency. Compounds with one NO ligand, such as mononitrosyl iron complexes (MNICs), have one NO stretching mode. Therefore, the basis spectrum with two absorption bands is assigned to the DNIC and that with one absorption band to the MNIC.

As mentioned previously, the basis spectrum with two red-shifted absorption bands at 1712 and 1617 cm^−1^ were assigned to [Fe(NO)_2_(MerS)_2_]^−*^. The red shift of the NO stretching modes indicates that the NO ligand in the excited state is more anionic than the ground state. The anionic character of NO is attributed to electron transfer from the iron center to the NO ligands, which is consistent with the result that [Fe(NO)_2_(MerS)_2_]^−*^ is an MLCT state. Various MLCTs have been identified in DNICs [31]. If the MLCT state leads to dissociation, NO^−^ could be released. Unfortunately, the excited electronic state of the metal complex cannot be readily calculated by the DFT method nor can the simple quantum calculation be utilized for the assignment. However, based on the shift in the vibrational frequencies of NO observed in the excited state and 70% recovery of the bleach signal, we concluded that the binding environment around the NO ligand in the excited DNIC first undergoes a change, followed by partial dissociation. The new binding environment in [Fe(NO)_2_(MerS)_2_]^−*^ can lead to the dissociation of either NO or NO^−^. The basis spectrum with one absorption band at 1753 or 1643 cm^−1^ must arise from the MNIC. The possible nascent MNIC compound involved in the photochemical reaction of [Fe(NO)_2_(MerS)_2_]^−^ is either [Fe(NO)(MerS)_2_] or [Fe(NO)(MerS)_2_]^−^, produced by the dissociation of NO^−^ or NO, respectively, from [Fe(NO)_2_(MerS)_2_]^−*^:

[Fe(NO)_2_(MerS)_2_]^−*^ → [Fe(NO)(MerS)_2_] + NO^−^

[Fe(NO)_2_(MerS)_2_]^−*^ → [Fe(NO)(MerS)_2_]^−^ + NO

The nascent photoproduct [Fe(NO)(MerS)_2_]^0,−^ can be immediately coordinated by the D_2_O solvent, resulting in [Fe(NO)(MerS)_2_(D_2_O)*_x_*]^0,−^ (where *x* = 1–3) [34]. In other words, [Fe(NO)(MerS)_2_(D_2_O)*_y_*]^0,−^ is in equilibrium with D_2_O-coordinated compounds:

[Fe(NO)(MerS)_2_(D_2_O)*y*]^0,−^ + D_2_O ⇌ [Fe(NO)(MerS)_2_(D_2_O)*_y+_*_1_]^0,−^

where *y* = 0–2. Among [Fe(NO)(CysS)_2_(H_2_O)*_x_*] with *x* = 1–3, [Fe(NO)(CysS)_2_(H_2_O)] was suggested to be the most abundant compound, where CysS is a deprotonated cysteine, because it was calculated to be the most stable compound [34]. To determine the most abundant compound among [Fe(NO)(MerS)_2_(D_2_O)*_x_*]^0,−^ with *x* = 1–3, their energies were calculated (Table 1). DFT calculations showed that [Fe(NO)(MerS)_2_(D_2_O)_2_]^0,−^ is the most stable compound among [Fe(NO)(MerS)_2_(D_2_O)*_x_*]^0,−^ with *x* = 1–3. Therefore, it can be suggested that the observed spectrum is dominated by the spectrum of [Fe(NO)(MerS)_2_(D_2_O)_2_]^0,−^. Hence, the compound with the 1643-cm^−1^ band can be assigned to either [Fe(NO)(MerS)_2_(D_2_O)_2_] or [Fe(NO)(MerS)_2_(D_2_O)_2_]^−^. As shown in Table 1, the calculated vibrational frequencies of the NO stretching mode in both [Fe(NO)(MerS)_2_(D_2_O)_2_] and [Fe(NO)(MerS)_2_(D_2_O)_2_]^−^ are consistent with the observed band at 1643 cm^−1^. Thus, the position of the observed band alone cannot differentiate [Fe(NO)(MerS)_2_(D_2_O_2_)] from [Fe(NO)(MerS)_2_(D_2_O)_2_]^−^.

As we use 10-fold equivalent of thiolates for sample preparation (see Section 3.1), the sample solution contains 9-fold extra thiolates. Because the extra thiolate present in the sample solution can coordinate to [Fe(NO)(MerS)_2_(D_2_O)_2_]^0,−^, [Fe(NO)(MerS)_3_(D_2_O)_2_]^−,2−^ can be produced too. The resulting compound is in equilibrium with D_2_O-coordinated compounds:

[Fe(NO)(MerS)_3_(D_2_O)*z*]^−,2−^ + D_2_O ⇌ [Fe(NO)(MerS)_3_(D_2_O)_*z*+__1_]^−,2−^

where *z* = 0–1. As shown is Table 1, [Fe(NO)(MerS)_3_]^−^ or [Fe(NO)(MerS)_3_(D_2_O)]^2−^ was determined to be the most stable compound among [Fe(NO)(MerS)_3_(D_2_O)*_y_*]^−,2−^ with *y* = 0–2. Thus, the 1753-cm^−1^ band arises from either [Fe(NO)(MerS)_3_]^−^ or [Fe(NO)(MerS)_3_(D_2_O)]^2−^. However, the calculated vibrational frequency of the NO stretching mode for [Fe(NO)(MerS)_3_]^−^ is consistent with the observed value, while that for [Fe(NO)(MerS)_3_(D_2_O)]^2−^ is not. Consequently, the band at 1753 cm^−1^ was assigned to the NO stretching mode of [Fe(NO)(MerS)_3_]^−^, not to that of [Fe(NO)(MerS)_3_(D_2_O)]^2−^.

As mentioned earlier, the observed band at 1643 cm^−1^ could not be assigned to any molecule using only the calculated vibrational frequencies of [Fe(NO)(MerS)_2_(D_2_O_2_)] and [Fe(NO)(MerS)_2_(D_2_O)*_2_*]^−^ that are expected to be produced upon the dissociation of NO^−^ or NO, respectively, from [Fe(NO)_2_(MerS)_2_]^−*^. Therefore, the following two kinetic schemes were considered to explain the photochemical reaction of excited [Fe(NO)_2_(MerS)_2_]^−^:

(Scheme 1): NO^−^ dissociation

[Fe(NO)_2_(MerS)_2_]^−*^ → [Fe(NO)(MerS)_2_] + NO^−^

[Fe(NO)(MerS)_2_] + 2D_2_O → [Fe(NO)(MerS)_2_(D_2_O)_2_] (**I-1**), Δ*G*_r_ = −7.7 kcal/mol

[Fe(NO)(MerS)_2_(D_2_O)_2_] + RS^−^ → [Fe(NO)(MerS)_3_(D_2_O)_2_]^−^, Δ*G*_r_ = −5 kcal/mol

[Fe(NO)(MerS)_3_(D_2_O)_2_]^−^ → [Fe(NO)(MerS)_3_]^−^ (**I-2**) + 2D_2_O, Δ*G*_r_ = −21.4 kcal/mol

(Scheme 2): NO dissociation

[Fe(NO)_2_(MerS)_2_]^−*^ → [Fe(NO)(MerS)_2_]^−^ + NO

[Fe(NO)(MerS)_2_]^−^ + 2D_2_O → [Fe(NO)(MerS)_2_(D_2_O)_2_]^−^ (**I′-1**), Δ*G*_r_ = −1.8 kcal/mol

[Fe(NO)(MerS)_2_(D_2_O)_2_]^2−^ + RS^−^ → [Fe(NO)(MerS)_3_(D_2_O)_2_]^2−^, Δ*G*_r_ = 4.3 kcal/mol

[Fe(NO)(MerS)_3_(D_2_O)_2_]^2−^ → [Fe(NO)(MerS)_3_(D_2_O)]^2−^ (**I′-2**) + D_2_O, Δ*G*_r_ = −13.5 kcal/mol

**Figure 6:**
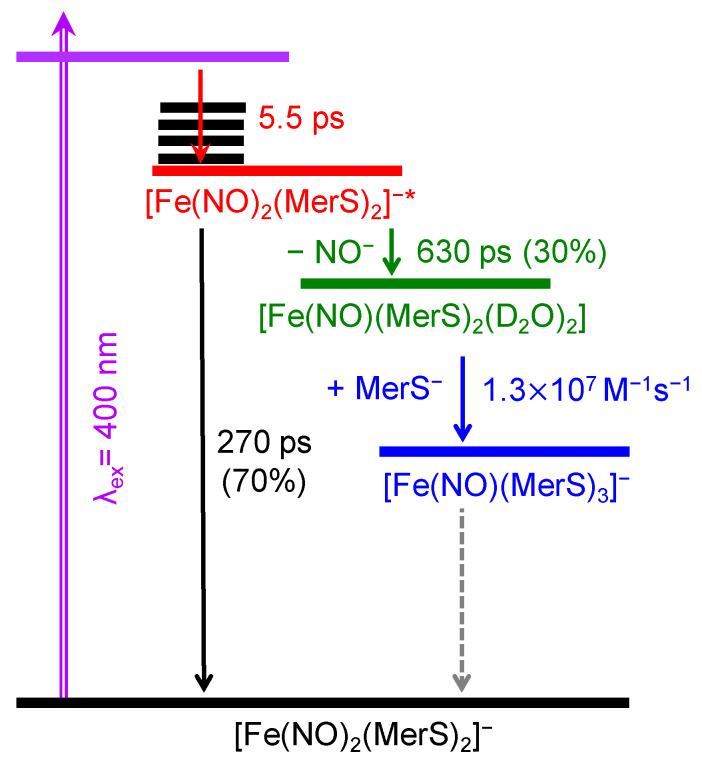
Schematics for the photoexcitation dynamics of [Fe(NO)_2_(MerS)_2_]^−^ in D_2_O at 293 K after excitation at 400 nm.

Here, Δ*G*_r_ is the calculated reaction Gibbs free energy. The overall reactions are exogenic. Both schemes produced two MNICs each; one MNIC decays to produce another MNIC as observed in the experiment. The observed vibrational frequencies at 1643 and 1753 cm^−1^ may arise from the NO stretching mode of the first and second MNICs, respectively. In other words, the band at 1643 cm^−1^ should be assigned to **I-1** or **I′-1**, and that at 1753 cm^−1^ to either **I-2** or **I′-2**. The calculated vibrational frequencies of **I-2** and **I′-2** show that the band at 1753 cm^−1^ should be assigned to **I-2**, indicating that Scheme 2 cannot produce the second MNIC with the 1753-cm^−1^ band observed in the current experiment. Consequently, the experimental observation is consistent with the dissociation of NO^−^ from [(MerS)_2_Fe(NO)_2_]^−*^, but not that of NO. Thus, the dissociation of NO from [(MerS)_2_Fe(NO)_2_]^−*^ is unlikely to occur in the photoexcitation of [(MerS)_2_Fe(NO)_2_]^−^. This observation is consistent with the report that NO does not dissociate from cysteine-ligated mononuclear DNICs upon excitation at 400 nm [16]. Therefore, Scheme 1 was used to describe the photoexcitation dynamics of [(MerS)_2_Fe(NO)_2_]^−^. Based on Scheme 1, the schematics for the photoexcitation dynamics of [(MerS)_2_Fe(NO)_2_]^−^ in D_2_O at 293 K after excitation at 400 nm is summarized in Figure 6.

According to the scheme shown in Figure 6, upon excitation at 400 nm, approximately 70% of the excited [Fe(NO)_2_(MerS)_2_]^−^ relaxes to the ground state with a time constant of 270 ps. The remaining compounds undergo NO^−^ dissociation, producing [Fe(NO)(MerS)_2_]^−^. The nascent photoproduct [Fe(NO)(MerS)_2_]^−^ first coordinated with a solvent molecule and then underwent a bimolecular reaction with thiolate with a rate constant of 1.3 × 10^7^ M^−1^s^−1^ taking place in a time constant of 150 ns in our experimental condition, producing [Fe(NO)(MerS)_3_]^−^—a more stable MNIC. The reaction proceeds much slower than the calculated diffusion-limited reaction with a rate constant of 6.5 × 10^9^ M^−1^s^−1^.

The formation mechanism of DNICs and RSE from Fe(II), NO, and RSH in aqueous solution was reported as follows [16,34,35]:

(Mechanism I)

Fe^2+^ + NO + 2RSH ⇌ [Fe^II^(NO)(RS)_2_] (**C**) + 2H^+^ (1st phase)

**C** → [Fe^I^(NO)(RS)] (**D**) + RS^•^ (2nd phase)

**D** + RSH + NO → DNIC + H^+^ ⇌ ½ RSE + RSH

According to Mechanism I, **C** is formed reversibly by the reaction of the Fe^II^ center with NO and two thiols. The equilibrium in the 1st phase is pH dependent; the concentration of **C** is high and the formation of **C** is faster at higher pH. Using thiol compounds such as glutathione and cysteine as well as small peptides containing cysteine (WCGPC and WCGPY), Ford et al. showed that the first stage proceeds in a few tens of milliseconds, while the unimolecular 2nd stage is the rate-limiting step with a time constant of a few seconds [34]. Their transient EPR data showed that intermediate **D** is in the triplet state [34,35]. Although not specifically shown, the remaining coordination sites of the intermediates could be occupied by the solvent (i.e., H_2_O). One H_2_O-coordinated [Fe^II^(NO)(RS)_2_] was calculated to be most stable complex [34]. Thus, **C** represents [Fe^II^(NO)(RS)_2_(H_2_O)] in the triplet state.

Interestingly, **I-1**, one of the intermediates in Scheme 1, is the most stable solvent-coordinated [Fe^II^(NO)(MerS)_2_], corresponding to species **C** in the formation mechanism of DNICs. Thus, according to the formation mechanism, the Fe(II) center of **I-1** can undergo spontaneous reduction producing intermediate **D** and a thiyl radical RS^•^. In other words, the reduction at the Fe(II) center of **I-1** competes with the coordination reaction with MerS^−^. While the reduction of the Fe(II) center in **I-1** takes a few seconds [34], the coordination of MerS^−^ to **I-1** occurs much faster with a rate constant of 1.3 × 10^7^ M^−1^s^−1^, taking place in a time constant of 150 ns in our experimental condition. Therefore, **I-1** reacts with MerS^−^, producing **I-2** instead of undergoing reduction at its Fe center in the current experimental conditions. If the concentration of RS^−^ ([RS^−^]) is small, the coordination of RS^−^ to **I-1** can be slower than the reduction of the Fe(II) center in **I-1**. When [**I-1**] × [RS^−^] > 10^−6^ M where [**I-1**] is the concentration of **I-1**, the MerS^−^ coordination to **I-1** proceeds with the initial rate of 13 Ms^−1^, faster than the reduction of the Fe(II) center (which takes a few seconds), resulting in **I**-**2**. Because **I**-**2** can be a dominant compound in the formation of DNICs, the possibility of the reduction of the Fe(II) center in **I-2** instead of **I**-**1** was also considered. The calculated Gibbs energies of the reduction of the Fe(II) center and the subsequent reaction in **I-2** and **I-1** are as follows (see Table 1).

[Fe(NO)(MerS)_3_]^−^ (**I-2**) → [Fe(NO)(MerS)_2_]^−^ + RS^•^, ΔG_r_ = −7.7 kcal/mol

[Fe(NO)(MerS)_2_]^−^ + NO → [Fe(NO)_2_(MerS)_2_]^−^, ΔG_r_ = −30.1 kcal/mol

[Fe(NO)(MerS)_2_(D_2_O)_2_] (**I-1**) → [Fe(NO)(MerS)(D_2_O)_2_] + RS^•^, ΔG_r_ = −11.6 kcal/mol

[Fe(NO)(MerS)(D_2_O)_2_] + RS^−^ + NO → [Fe(NO)_2_(MerS)_2_]^−^ + 2D_2_O, ΔG_r_ = −52.6 kcal/mol

The reductions of Fe centers in **I-2** and **I-1** are exogenic and thus readily proceed spontaneously. Unless [**I-1**] × [MerS^−^] << 10^−8^ M, the formation of **I-2** is faster than the Fe center reduction in **I-1**; in addition, the Fe(II) center of **I-2** undergoes the reduction. Although the formation mechanism in Mechanism 1 describes the reduction of **C**, the mononitrosyl intermediate with the reduced Fe center, Fe(I), had not been specified, although it been attributed to either [Fe^I^(NO)(RS)] or [Fe^I^(NO)(RS)_2_]^−^ [34,35]. Our experiment shows the presence of **I-2**, which has a reducible Fe(II) center, which reveals that the reduced compound with Fe(I) can be [Fe^I^(NO)(RS)_2_]^−^. Therefore, the formation mechanism of DNICs and RSE from Fe(II), NO, and RSH in aqueous solution can be shown as follows:

(Mechanism 2)

Fe^2+^ + NO + 2RSH ⇌ [Fe^II^(NO)(RS)_2_] (**C**) + 2H^+^ (1st phase)

**C** + RS^−^ → [Fe^II^(NO)(RS)_3_]^−^ (**C′**) (1st phase)

**C′** → [Fe^I^(NO)(RS)_2_]^−^ (**D′**) + RS^•^ (2nd phase)

**D′** + NO → DNIC + H^+^ ⇌ ½ RSE + RSH

In Mechanism 2, **C′** instead of **C** undergoes the reduction in the Fe center. If [**C**] × [RS^−^] << 10^−8^ M, where the coordination reaction is too slow, the reduction of **C** would be faster and would occur through Mechanism 1. Therefore, we suggest that the formation mechanism depends on [RS^−^]: Mechanism 2 works in high [RS^−^] and Mechanism 1 in low [RS^−^].

## 3. Materials and Methods

### 3.1. Sample Preparation

FeSO_4_(H_2_O)_7_ and 2-mercaptoethanol were purchased from Sigma-Aldrich (St. Louis, MO, USA). NO gas was purchased from PSG Dover (Oak Brook, IL, USA). To ensure the elimination of dissolved oxygen, all solutions were thoroughly deoxygenated by bubbling nitrogen gas through them. The NO gas was immediately purified before use by passing it through a 3-M NaOH solution, which effectively removed NO_2_ and N_2_O_3_ produced by reactions with O_2_ in the NO introduction line. A 60-mM solution of a mononuclear anionic DNIC with two 2-mercaptoethanol ligands, [(HOCH_2_CH_2_S)_2_Fe(NO)_2_]^−^, was synthesized by first dissolving an appropriate amount of FeSO_4_(H_2_O) in D_2_O. Excess purified NO was then injected into this solution, followed by the addition of a 10-fold equivalent of 2-mercaptoethanol to the NO-saturated Fe(II) solution. The pH of the resulting solution was adjusted to be basic (pD = 10.5) using a 10-M NaOH solution, and the solution was maintained in a NO-saturated state by further injecting the purified NO. The successful synthesis of the DNIC was checked by collecting electronic (UV-3100, Sinco, Seoul, Republic of Korea) and FTIR (Spectrum 3, Perkin Elmer, Shelton, CA, USA) spectra of the final product. The synthesis of DNIC was confirmed by identifying the characteristic peaks in the electronic and vibrational spectra [16,26,27,28,29,30], as illustrated in Figure 1. D_2_O was used to avoid the absorption of H_2_O away from the spectral region of interest.

### 3.2. Time-Resolved Infrared (IR) Spectroscopy

The details of the TRIR spectrometer have been previously described elsewhere [32,36,37,38,39,40]. In brief, a commercial Ti:sapphire oscillator/amplifier system (Spitfire Ace, Spectra Physics, Milpitas, CA, USA) generated 100-fs pulses at 800 nm with an energy of 700 μJ at a repetition rate of 2 kHz. A portion of these amplified pulses was directed to a home-built optical parametric amplifier (OPA) to produce near-IR signal and idler pulses. The near-IR pulses from the OPA were then difference frequency mixed in a 1-mm-thick, type-Ⅰ AgGaS_2_ crystal to yield tunable mid-IR probe pulses (120 fs, 160 cm^−1^ spectral width). The remaining 800-nm pulses were frequency doubled in a type-I, 0.6-mm-thick β-barium borate crystal, resulting in a 140-fs, 400-nm pulse with the energy of 10 μJ, a portion of which was used as the pump pulse to excite the sample. A translational stage was utilized to adjust the optical delay between the pump and the probe pulses, accommodating delays of up to 1 ns. As the optical delay provided by this method becomes impractically long and extends over a few nanoseconds, the femtosecond pump pulse was replaced with a commercial nanosecond tunable laser, operated by an optical parametric oscillator (NT240, EKSPLA, Vilnius, Lithuania) for longer delays of over 1 ns. Synchronization of the nanosecond pump pulse with the femtosecond mid-IR probe pulse was achieved using an electronic digital delay generator (DG535, Stanford Research Systems, Sunnyvale, CA, USA). The instrument response function was approximately 0.2 ps for the femtosecond pump pulse and 2.5 ns for the nanosecond pump pulse. To acquire the isotropic absorption spectrum, the polarization of the pump pulse was oriented at the magic angle (54.7°) relative to the probe pulse. To mitigate nonlinear effects associated with a high pump intensity, the pump pulse energy was set at approximately 2 μJ, with a beam diameter of 230 μm. The mid-IR probe pulse with the energy of ~10 nJ and a beam diameter of 200 μm passed through the sample excited by the 400-nm pump pulse. The resultant mid-IR pulse, containing sample information, was dispersed using a 320-mm IR monochromator (IHR320, Horiba, Piscataway, NJ, USA) equipped with a 150 L/mm grating and a N_2_(l)-cooled 1 × 128 HgCdTe array detector (Infrared Associates, Stuart, FL, USA), achieving a spectral resolution of ~1.3 cm^−1^/pixel at 1760 cm^−1^. The probe pulse was centered at 1760, 1720, 1680, or 1630 cm^−1^ to facilitate probing over a broader spectral region than that afforded by the 160-cm^−1^ spectral width of the pulse.

### 3.3. Computational Details

Gaussian 16 software package was used for the quantum mechanical calculation of molecular geometry optimization and vibrational frequency analysis using the DFT method with TPSSh/def2SVP [41,42,43]. The TPSSh functional was chosen because this hybrid meta-GGA has been benchmarked for transition-metal-containing diatomic systems, showing improved agreement with experimental bond lengths and energies compared to other common functionals [44]. The TPSSh functional with the def2SVP basis set was employed without an additional empirical dispersion correction. This choice follows prior computational studies on nitrosyl complexes that adopted the same level of theory to reproduce experimental geometries and vibrational frequencies [44]. Water solvent was incorporated using the polarized continuum model (PCM) [45]. PCM was chosen because it provides a computationally efficient and reliable description of aqueous solvation for transition-metal complexes, and it has been widely employed in related Fe–NO computational studies. In addition, explicit solvation was considered by coordinating D_2_O molecules to key intermediates, which allows the treatment of specific solvent–metal interactions beyond the implicit PCM description. As [Fe(NO)_2_(MerS)_2_]^−^ is in a doublet state, spin unrestricted level of theory without symmetry constraints was used. For the sake of simplicity, all calculations were performed using methyl thiol-substituted DNIC (Mt-DNIC) instead of [Fe(NO)_2_(MerS)_2_]^−^. Photodeligation of one NO^−^ resulted in a radical species that is deficient in one NO^−^, i.e., [Fe(NO)(RS)_2_]. The D_2_O solvent can coordinate to the empty positions of [Fe(NO)(RS)_2_] forming [Fe(NO)(RS)_2_(D_2_O)*_x_*], *x* = 1–3 [34]. The intermediate can further react with the abundant thiolate in solution producing [Fe(NO)(RS)_3_]^−^. The possible intermediates and products were also determined. The scaling factor was (0.91–0.966) adjusted to match the calculated vibrational frequencies of the Mt-DNIC with the positions of the bands forming the eigenfunctions used to fit the TRIR of [Fe(NO)_2_(MerS)_2_]^−^.

## 4. Conclusions

Detailed photoexcitation dynamics of [Fe(NO)_2_(MerS)_2_]^−^ in D_2_O after excitation at 400 nm was probed by using femtosecond TRIR spectroscopy. Photoexcitation at 400 nm resulted in [Fe(NO)_2_(MerS)_2_]^−^ in the MLCT excited state. Approximately 70% of the excited [Fe(NO)_2_(MerS)_2_]^−^ relaxed to the ground state with a time constant of 270 ps; the remaining dissociated NO^−^, producing the nascent [Fe(NO)(MerS)_2_] with a time constant of 630 ps. Although the nascent product could undergo reduction at the Fe center producing a MerS^•^ radical and [Fe(NO)(MerS)], the intermediate during the formation of [Fe(NO)_2_(MerS)_2_]^−^, it quickly reacted bimolecularly with abundant MerS^−^, producing [Fe(NO)(MerS)_3_]^−^ with a rate constant of 1.3 × 10^7^ M^−1^s^−1^. [Fe(NO)(MerS)_3_]^−^ could also undergo reduction at the Fe center, producing MerS^•^ radical and [Fe(NO)(MerS)_2_]^−^, which readily became [Fe(NO)_2_(MerS)_2_]^−^ by reacting with NO in solution. This work represents an important step forward in improving the current understanding of the photochemical properties of DNICs and provides a detailed kinetic framework for photochemical pathways. The ultrafast dynamics of the dissociation of the NO^−^ anion and the formation of stable coordination products have important implications for the design of NO emitters in biological systems. These findings pave the way for future studies to optimize the photophysical properties of DNICs and expand their potential in biomedical research.

## Figures and Tables

**Table 1:** Calculated energies and vibrational frequencies of NO for the compounds related to the photoexcitation dynamics of [Fe(NO)_2_(MerS)_2_]^−^ in D_2_O at 293 K after excitation at 400 nm using the DFT method with TPSSh/def2svp. For the sake of simplicity, all the calculation was performed using methyl thiol. Δ*G* is the reaction Gibbs free energy to produce the corresponding compound from [Fe(NO)_2_(MerS)_2_]^−^. As triplet intermediates were observed in the formation mechanism of [Fe(NO)_2_(RS)_2_]^−^ [30,34], only triplet compounds were considered here. Considering singlet compounds did not affect the conclusion.

Compound	Spin	ν_1_ (cm^−1^)	ν_2_ (cm^−1^)	Δ*G* (kcal/mol)	*G* (Hartree)
[Fe(NO)_2_(MerS)_2_]^−^	doublet	1839.75	1788.88	0	−2399.38
[Fe(NO)(MerS)_2_]	doublet	1899.48		71.9	−2269.37
[Fe(NO)(MerS)_2_(D_2_O)]	doublet	1870.24		66.8	−2345.74
[Fe(NO)(MerS)_2_(D_2_O)_2_]	doublet	1806.56		64.2	−2422.11
[Fe(NO)(MerS)_2_(D_2_O)_3_]	doublet	1788.96		84.4	−2498.44
[Fe(NO)(MerS)_2_]^−^	triplet	1772.29		30.1	−2269.53
[Fe(NO)(MerS)_2_(D_2_O)]^−^	triplet	1753.69		31.1	−2345.89
[Fe(NO)(MerS)_2_(D_2_O)_2_]^−^	triplet	1732.24		28.3	−2422.26
[Fe(NO)(MerS)_2_(D_2_O)_3_]^−^	triplet			Not converged	
[Fe(NO)(MerS)_3_]^−^	doublet	1812.75		37.8	−2707.49
[Fe(NO)(MerS)_3_(D_2_O)]^−^	doublet	1737.64		41.9	−2783.84
[Fe(NO)(MerS)_3_(D_2_O)_2_]^−^	doublet	1731.08		59.2	−2860.18
[Fe(NO)(MerS)_3_]^2−^	triplet	1669.39		19.8	−2707.56
[Fe(NO)(MerS)_3_(D_2_O)]^2−^	triplet	1661.53		19.1	−2783.92
[Fe(NO)(MerS)_3_(D_2_O)_2_]^2−^	triplet	1671.19		32.6	−2860.27
[Fe(NO)(MerS)]	triplet	1821.47		57.5	−1831.42
[Fe(NO)(MerS)(D_2_O)]	triplet	1814.10		52.5	−1907.80
[Fe(NO)(MerS)(D_2_O)_2_]	triplet	1775.38		52.6	−1984.16
NO^−^	triplet				−129.90
NO^−^	singlet				−129.84
NO	doublet				−129.81
MerS	doublet				−437.92
MerS^−^	singlet				−438.06
D_2_O	singlet				−76.36

## Data Availability

Data are contained within the article.

## Abbreviations

The following abbreviations are used in this manuscript:

DNIC	Dinitrosyl iron complex
NO	Nitric oxide
MNIC	Mononitrosyl iron complex
RRE	Roussin’s red ester
TRIR	Time-resolved infrared
EPR	Electron paramagnetic resonance
DFT	Density functional theory
MLCT	Metal-to-ligand charge transfer
SVD	Singular value decomposition
IR	Infrared
OPA	Optical parametric amplifier
PCM	Polarized continuum model
